# An Interpretable Multimodal Machine-Learning Model for Non-Invasive Preoperative Glioma Grading

**DOI:** 10.3390/cancers18081204

**Published:** 2026-04-10

**Authors:** Xianfeng Rao, Min Yang, Hao Chen, Guanhao Li, Li Wu, Liudong Gong, Mingchun Yang, Haiyang Wang, Ye Ding, Guanxi Chen, Xianjun Rao, Na Zhang, Xiaoxiong Wang, Lei Teng

**Affiliations:** 1 Department of Neurosurgery, The First Affiliated Hospital of Harbin Medical University, Harbin 150001, China; 2 Department of Pediatric Surgery, The Sixth Affiliated Hospital of Harbin Medical University, Harbin 150023, China; 3 Department of Spleen and Stomach Diseases, Wangjing Hospital, China Academy of Chinese Medical Sciences, Beijing 100102, China; 4 Department of Laboratory Diagnostics, The First Affiliated Hospital of Harbin Medical University, Harbin 150001, China

**Keywords:** glioma, preoperative grading, machine learning, predictive model, magnetic resonance spectroscopy

## Abstract

**Background**: Gliomas are the most common primary malignant tumors of the central nervous system. Accurate preoperative grading is essential for individualized surgical planning and treatment selection; however, reliable non-invasive prediction tools integrating multimodal preoperative data remain limited. This study aimed to develop and internally validate an interpretable machine-learning model for non-invasive glioma grading. **Methods**: Clinical and imaging data from 400 patients with pathologically confirmed gliomas were retrospectively collected. Twenty-four preoperative variables were analyzed. The dataset was randomly divided into training and validation cohorts (7:3). Feature selection was performed using a combination of the Boruta algorithm and logistic regression analyses, followed by correlation filtering. Seventeen machine-learning algorithms were benchmarked using five-fold cross-validation, and the optimal model was evaluated in the independent validation cohort using ROC analysis, calibration assessment, precision–recall curves, and decision curve analysis. Model interpretability was examined using SHAP. **Results**: Eight key predictors were identified, including age, focal neurological deficits, midline shift, tumor laterality, tumor lobar location, enhancing tumor volume, and MRS-derived Cho/NAA and Cho/Cr ratios. The Random Forest model achieved an area under the ROC curve of 0.946 (95% CI: 0.902–0.989) in the validation cohort. Calibration analysis demonstrated reasonable agreement between predicted and observed outcomes, and the precision–recall curve yielded an average precision of 0.98. Decision curve analysis indicated net clinical benefit across relevant probability thresholds. **Conclusions**: A multimodal machine-learning model integrating clinical, structural imaging, and MRS-derived metabolic features was developed and internally validated for non-invasive preoperative glioma grading. The model showed good discrimination and calibration and provided individualized probability estimates, suggesting potential value for preoperative risk stratification. However, clinical deployment remains premature, and further external validation is required.

## Simple Summary

Glioma grading before surgery is essential because treatment planning and expected outcomes differ greatly between low-grade and high-grade tumors. In routine practice, grading often depends on postoperative pathology, while preoperative imaging and symptoms alone may be uncertain. We developed and internally validated a machine-learning model that combines readily available preoperative information, including clinical symptoms, standard imaging features, and metabolic measurements from magnetic resonance spectroscopy. Using data from 400 patients, the model provided an individualized probability of high-grade glioma and showed strong performance in an independent validation group. To improve transparency, we used an explanation method to show how each factor contributed to the prediction. This approach may help clinicians better stratify risk before surgery, support discussions with patients, and guide planning of surgical strategies and postoperative management. Further validation in external cohorts is needed before broader clinical adoption.

## 1. Introduction

Glioma is the most common primary malignant intracranial tumor in adults, accounting for approximately 80% of central nervous system malignancies [1,2]. According to the World Health Organization (WHO) classification, gliomas are categorized into low-grade (LGG, grades I–II) and high-grade (HGG, grades III–IV) tumors [3,4]. Compared with LGG, HGG exhibits aggressive growth, treatment resistance, and markedly poorer survival outcomes [5,6,7,8,9,10]. Therefore, accurate preoperative grading is essential for determining surgical extent, intraoperative functional mapping strategies, and postoperative adjuvant therapy.

Currently, glioma grading relies on postoperative histopathology combined with molecular markers such as IDH and TP53 [3]. However, this gold standard is invasive and only available after surgery. Although stereotactic biopsy may assist preoperative evaluation, sampling errors due to intratumoral heterogeneity limit its reliability [11]. In clinical practice, grading is primarily based on contrast-enhanced MRI, yet enhancement patterns are not always specific: some HGGs exhibit minimal enhancement, while certain LGGs present atypical enhancement features, leading to potential misclassification [12]. These limitations highlight the need for more reliable non-invasive preoperative assessment tools.

To improve diagnostic performance, various approaches have been explored. Radiomics-based models derived from multiparametric MRI have shown promising results [13,14,15], and clinical predictors have also been investigated [16]. In addition, functional MRI techniques such as magnetic resonance spectroscopy (MRS), diffusion tensor imaging (DTI), and amide proton transfer (APT) imaging provide complementary metabolic and microstructural information [17,18,19]. However, most previous studies rely predominantly on imaging features alone, with limited integration of routinely available clinical symptoms and metabolic biomarkers. Moreover, the combined utilization of multimodal data in a clinically accessible and standardized manner remains insufficient, and the generalizability and interpretability of existing models are still limited.

Artificial intelligence and machine-learning approaches have rapidly advanced in medical imaging and clinical decision support, including applications in neuro-oncology [20]. Recent studies have increasingly focused on deep learning frameworks and data augmentation strategies. For example, generative adversarial networks (GANs), such as Pix2Pix-based models, have been applied to generate synthetic MRI data to address data scarcity and improve classification performance [21]. Similarly, conditional deep convolutional neural networks combined with GAN-generated datasets have demonstrated promising results in brain tumor image classification [22]. However, successful translation into neurosurgical practice requires not only predictive performance but also interpretability and biological plausibility.

To address these gaps, we developed an interpretable multimodal machine-learning model for preoperative glioma grading. The framework integrates three categories of routinely accessible data: clinical symptoms, structural imaging indicators (midline shift and enhanced tumor volume), and MRS-derived metabolic ratios. Through rigorous feature selection and systematic algorithm comparison, we aimed to construct a high-performance yet transparent model for exploratory evaluation in a single-center cohort. This study aims to assess the feasibility and potential value of integrating multimodal data and interpretable machine-learning methods for preoperative glioma grading.

## 2. Materials and Methods

### 2.1. Study Population

This study retrospectively collected cases pathologically confirmed glioma following surgery at the hospital between January 2019 and December 2024.

The inclusion criteria were: (1) age ≥ 18 years; (2) availability of a definitive postoperative histopathological diagnosis; (3) availability of complete preoperative imaging datasets, including cranial CT, conventional MRI sequences (non-contrast and contrast-enhanced T1-weighted sequences), and MRS data. Exclusion criteria included: (1) absence of key clinical information; (2) incomplete imaging data or the presence of severe artifacts affecting analysis; (3) concomitant neurological disorders or other primary intracranial malignant tumors; (4) a history of prior surgical intervention for glioma before admission. All included patients had complete data for the 24 prespecified candidate predictors. Therefore, no imputation was required for model development and no missing values were present in the final analytic dataset (the default imputation component in the machine-learning pipeline was retained for workflow consistency but did not modify any values). All included patients were evaluated and managed by a neurosurgical team in accordance with relevant clinical guidelines. The study protocol was approved by the Ethics Committee, with informed consent waived.

### 2.2. Data Collection

Data were obtained through a systematic review of electronic medical records and the imaging archiving system. Collected variables included: 1. Demographics and baseline status: age, sex, history of smoking and alcohol consumption, and comorbidities (hypertension, diabetes). 2. Clinical presentation: Karnofsky Performance Status (KPS) score at admission, duration of symptoms, and the presence of specific neurological symptoms, categorized as follows: a. Seizure—any type of preoperative seizure event; b. Intracranial increased pressure (IIP)—presence of at least one of the following: headache, nausea, vomiting, or visual blurring associated with papilledema; c. Focal neurological deficits (FND)—objective signs including motor impairment (monoparesis or hemiparesis with strength ≤ grade 4), sensory disturbances, language impairment (aphasia or dysarthria), or visual field defects; d. Cognitive impairment (CI)—such as memory decline, slowed responsiveness; e. Cerebellar symptoms (CS)—including ataxia or gait instability. 3. Imaging features: MS on CT, measured at the axial slice demonstrating the maximal tumor diameter, with displacement ≥ 2 mm considered positive. Tumor location was recorded as laterality (left hemisphere, right hemisphere, or crossing the midline) and involved lobe(s) (multilobar, frontal, temporal, parietal, occipital lobe, deep structure). ETV was estimated on contrast-enhanced T1-weighted MRI using the ellipsoid approximation: V = (a × b × c)/2, where a, b, and c represent the three maximal perpendicular diameters. This method was chosen because it reflects routine clinical practice and does not require dedicated segmentation software; however, it is an approximation and may introduce measurement error compared with manual or automated volumetry. For MRS, the voxel/region of interest was placed within the solid enhancing portion of the tumor while avoiding necrosis, hemorrhage, cystic areas, and surrounding normal tissue as much as possible; Cho/NAA, Cho/Cr, and NAA/Cr ratios were obtained. MRI and MRS examinations were performed using a 1.5-T scanner (Siemens Aera, Siemens Healthineers, Erlangen, Germany) equipped with a 24-channel head and neck coil. Conventional MRI acquisition included axial T1-weighted imaging (T1WI), fat-suppressed T2-weighted imaging (T2WI), and contrast-enhanced T1-weighted imaging, with representative parameters as follows: repetition time (TR) 383–2700 ms, echo time (TE) 11–78 ms, slice thickness 5 mm, interslice gap 1 mm, field of view 180–240 mm, and matrix size 256 × 180 to 384 × 308, depending on the sequence protocol. MRS was acquired using a single-voxel technique with a point-resolved spectroscopy (PRESS) sequence, with the voxel positioned within the solid tumor component while avoiding necrosis and surrounding normal tissue. The principal acquisition parameters were: repetition time (TR) 1500–2000 ms, echo time (TE) 135 ms, voxel size approximately 10 × 10 × 10 mm^3^ (1.0 cm^3^), and number of excitations 96–128. All therapeutic strategies were determined by neurosurgeons based on individual patient characteristics and in accordance with current clinical guidelines for glioma management. All imaging measurements (including MS and ETV) were performed by a radiologist with 10 years of neuroimaging experience who was blinded to the pathological grade.

### 2.3. Definition of HGG and LGG

The final diagnosis for all patients was established according to the integrated diagnostic framework of the 2021 WHO Classification of Tumors of the Central Nervous System (Fifth Edition). This diagnosis incorporates histological morphology with key biological indicators including IDH mutation status, TP53 status, and the Ki-67 proliferation index. Tumor grading in this study was based on this integrated diagnosis. Accordingly, WHO grades I and II were classified as low grade gliomas (LGG), while grades III and IV were classified as high grade gliomas (HGG).

### 2.4. Sample Size and Statistical Power

A total of 400 patients were enrolled and divided into a training set (n = 280) and an internal validation set (*n* = 120) in a 7:3 ratio using stratified sampling to preserve the HGG/LGG proportion. For reproducibility of resampling, model training, and hyperparameter optimization, a fixed random seed (random_state = 123) was applied throughout the machine-learning pipeline (SMOTETomek, Random Forest training, and OptunaSearchCV). The training set size exceeded the minimum required sample size (*n* = 240), calculated based on an events-per-variable (EPV) criterion of ≥10 for 24 candidate predictors. The validation cohort (*n* = 120) accounted for 30% of the total sample, consistent with commonly recommended proportions (20–30%) for internal validation [23,24], supporting stable and reliable performance estimation.

### 2.5. Statistical Analysis

Categorical variables were presented as frequencies and percentages, and comparisons between groups were performed using the χ^2^ test or Fisher’s exact test. The normality and homogeneity of variance of continuous variables were assessed using the Shapiro–Wilk test and Levene’s test, respectively. Variables conforming to a normal distribution were expressed as mean ± standard deviation and compared using the independent-samples *t* test, whereas non-normally distributed variables were expressed as median (interquartile range) and compared using the Mann–Whitney U test [25]. All statistical analyses and machine-learning procedures were performed using Python (version 3.10.14) with the following packages: scikit-learn (version 1.3.2), SHAP (version 0.42.1), and Optuna (version 3.4.0). Feature selection was conducted within the training set only. The Boruta algorithm and univariate/multivariate logistic regression were applied independently, and only predictors identified by both approaches (i.e., the intersection set) were retained. Subsequently, zero-variance features were removed and highly correlated predictors (|r| > 0.8) were excluded. Seventeen machine-learning algorithms were evaluated to cover a broad range of common model families, including linear models, Bayesian classifiers, instance-based methods, kernel-based methods, tree-based ensembles, and boosting algorithms. This design aimed to reduce algorithmic selection bias and identify the most robust approach for multimodal glioma grading. Model training and benchmarking were performed in the training set using 5-fold stratified cross-validation. For the selected model, hyperparameter optimization was further performed within the training set using OptunaSearchCV with stratified 10-fold cross-validation, with the area under the receiver operating characteristic curve (AUC) used as the optimization objective. The hyperparameter search space included key parameters such as the number of trees, maximum depth, and minimum samples split, which were optimized within predefined ranges. Key hyperparameters were automatically tuned within predefined search ranges to identify the optimal model configuration. Evaluation metrics included Accuracy, AUC, Recall, Precision, F1-score, Cohen’s Kappa, MCC, Log Loss, Brier score, and training time. The final model was then validated in the internal validation set using receiver operating characteristic (ROC) curves, calibration curves, precision–recall (PR) curves, and decision-curve analysis (DCA) to assess its discriminative ability, calibration, identification accuracy, and clinical utility. For classification-based metrics (accuracy, sensitivity, specificity, and confusion matrix), the optimal probability threshold for predicting HGG was determined based on the maximum F1-score during model evaluation, yielding a cutoff of P(HGG) = 0.38. To enhance interpretability, SHAP was used to visualize feature contributions via summary and dependence plots. For the final Random Forest model, SHAP values were computed using the tree-based explainer (shap.TreeExplainer; SHAP package version 0.42.1), which implements TreeSHAP for tree ensembles. For categorical variables, SHAP values reflect the contribution of specific one-hot encoded categories and were interpreted accordingly. The individual prediction example was selected from the validation set.

## 3. Results

### 3.1. Baseline Characteristics

**Figure 1:**
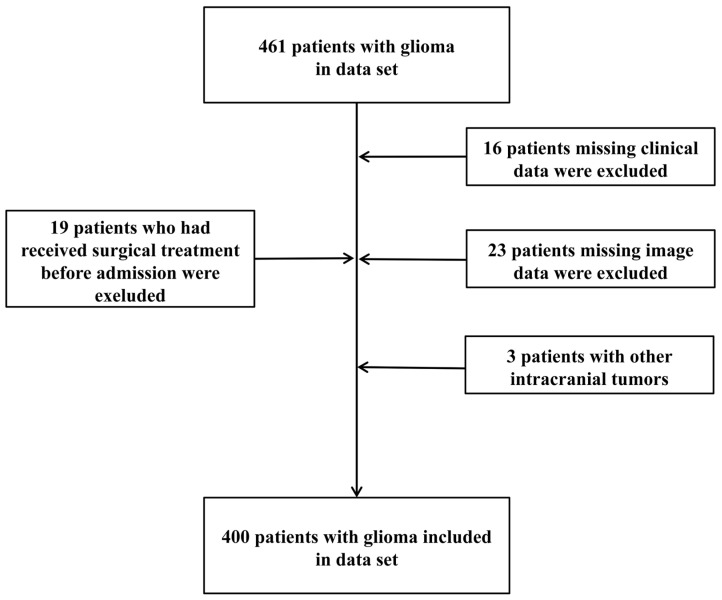
Flowchart of patient inclusion and exclusion. This flowchart illustrates the screening process used to derive the final study cohort from an initial pool of 461 patients with glioma. A total of 61 patients were excluded according to the prespecified criteria, and 400 patients with complete preoperative clinical informatiosn, imaging datasets, and postoperative pathological confirmation were included for analysis. This structured selection process provides a transparent overview of cohort construction.

A total of 461 patients with glioma were initially identified. After rigorous medical and imaging quality review, 61 cases were excluded due to incomplete clinical data, suboptimal imaging quality, or prior interventions, leaving 400 patients in the final cohort (Figure 1). The cohort was randomly divided into a training set (*n* = 280) for model development and an internal validation set (*n* = 120). Overall, 75% (*n* = 300) were HGG and 25% (*n* = 100) were LGG. Significant differences between the HGG and LGG groups were observed in multiple demographic, clinical, and imaging variables (Table 1). Specifically, HGG patients were older, had a lower incidence of seizures, presented with more frequent FND, and had a significantly higher proportion of MS. In terms of quantitative imaging characteristics, HGG patients exhibited significantly higher ETV, Cho/NAA, and Cho/Cr values compared to LGG patients.

### 3.2. Prediction Factor Selection

**Figure 2:**
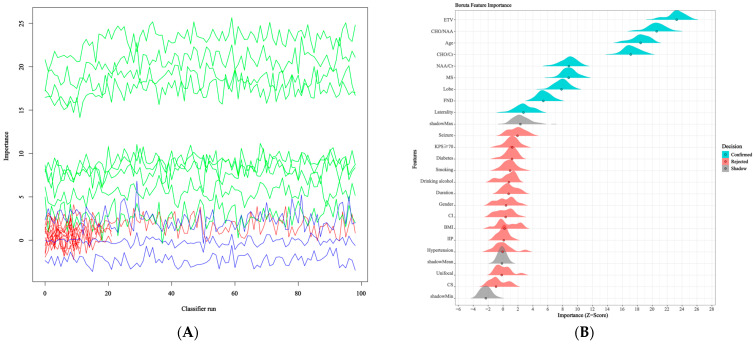

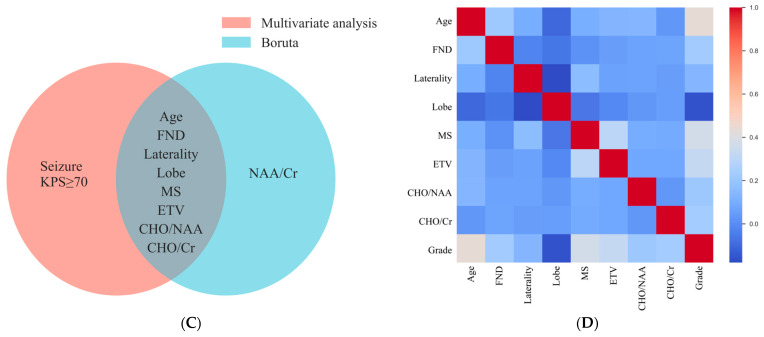
Feature selection process and results based on multimodal preoperative data. (**A**) Boruta feature importance across classifier runs. Variables consistently ranked above shadow features (green) were confirmed as important, while those below (blue) were rejected, indicating clear separation between relevant and non-informative predictors. Red bars represent shadow features used as a reference for importance comparison. (**B**) Distribution of Boruta importance scores (Z-scores). Variables such as ETV, CHO/NAA, Age, and CHO/Cr show higher median importance and more stable distributions, suggesting stronger and more consistent contributions to glioma grading. (**C**) Overlap of selected features from Boruta and regression analyses. The intersection highlights robust predictors (Age, FND, Laterality, Lobe, MS, ETV, CHO/NAA, and CHO/Cr) that were consistently identified across different methods, improving the reliability of feature selection. (**D**) Correlation heatmap of selected variables. Most feature pairs show weak to moderate correlations, with no evident strong multicollinearity, supporting the inclusion of these variables in the subsequent model.

Prediction factor selection was performed exclusively within the training cohort (*n* = 280) after the initial data split to prevent potential information leakage. The independent validation set was not involved in any feature selection or model development procedures. Univariate analysis identified several candidate variables significantly associated with glioma grading (*p* < 0.05), including age, seizures, FND, tumor laterality, lobar location, KPS ≥ 70, MS, ETV, and MRS indices (Cho/NAA, Cho/Cr, NAA/Cr). No significant differences were observed between the two groups in terms of basic characteristics such as gender, hypertension, diabetes, smoking, and alcohol history (Table 2). Variables with a *p* < 0.05 in univariate analysis were included in a multivariate logistic regression model (Table 3). Independent predictors for high-grade glioma were identified as: older age (OR = 1.08, 95% CI: 1.06–1.11), presence of FND (OR = 4.94, 95% CI: 2.25–10.86), MS (OR = 5.44, 95% CI: 2.88–10.27), larger ETV (OR = 1.03, 95% CI: 1.02–1.04), higher Cho/NAA (OR = 1.04, 95% CI: 1.01–1.07), higher Cho/Cr (OR = 1.20, 95% CI: 1.09–1.33). Conversely, the presence of seizures (OR = 0.26, 95% CI: 0.14–0.50), high KPS score (OR = 0.45, 95% CI: 0.23–0.87), and certain lobar locations (OR = 0.81, 95% CI: 0.67–0.91) were negatively associated with HGG. In parallel, the Boruta algorithm was applied within the training cohort to assess feature importance based on a random forest classifier, identifying 9 important features (Figure 2A,B). By intersecting the independent predictors from multivariate regression with the features selected by Boruta, we finalized a set of eight core preoperative variables for model construction (Figure 2C): age, FND, tumor laterality, tumor lobar location, MS, ETV, Cho/NAA ratio, and Cho/Cr ratio. Further Spearman correlation analysis revealed weak correlations between the variables (all |r| < 0.55), suggesting the absence of severe multicollinearity and supporting inclusion of these variables in subsequent model construction(Figure 2D).

### 3.3. Machine Learning Model Training and Selection

**Figure 3:**
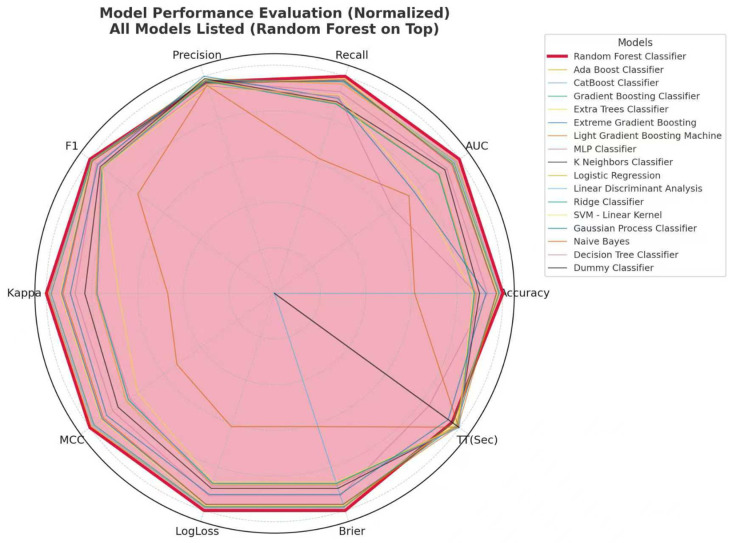
Performance comparison of multiple machine-learning classifiers in glioma grading prediction. This figure shows the normalized performance of 17 machine-learning models evaluated using multiple metrics, including Accuracy, AUC, Recall, Precision, F1-score, Cohen’s Kappa, MCC, Log Loss, Brier score, and training time. All metrics were normalized to enable cross-model comparison on a unified scale. Models are ordered according to their overall performance across the evaluated metrics. This comparative analysis facilitates the identification of models with balanced and robust performance across different evaluation criteria. The Random Forest classifier showed the highest overall performance among the evaluated models and was therefore selected as the final model for subsequent analysis.

Our model was based on the 8 features identified in the previous section. In the training set, 17 machine learning algorithms were benchmarked using 5-fold cross-validation. Model performance was evaluated using Accuracy, AUC, Recall, Prec., F1, Kappa, MCC, Log Loss, Brier, and TT(Sec). Comparative analysis showed that the Random Forest algorithm demonstrated the highest overall performance among the evaluated models, achieving the best AUC and consistently strong performance across multiple evaluation metrics (Supplementary Table S1) (Figure 3). Therefore, we selected the Random Forest model as the predicting model for preoperative glioma grading in this study.

### 3.4. Model Performance Validation

**Figure 4:**
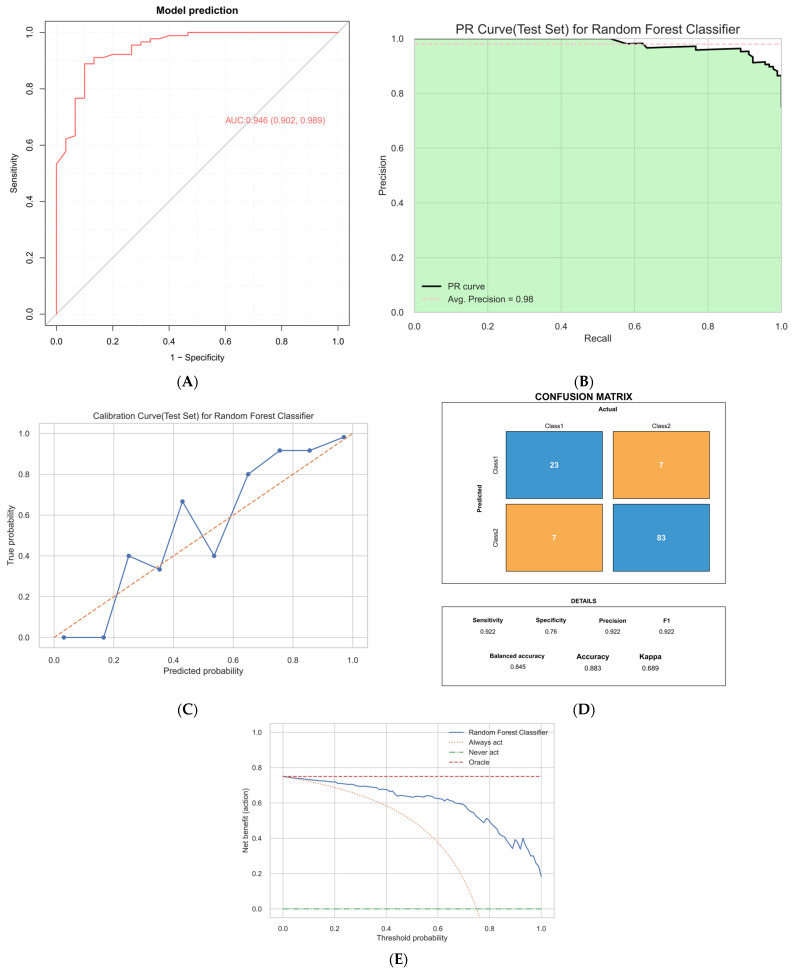
Performance evaluation of the random forest model in the internal validation set. (**A**) ROC curve showing high discriminative ability (AUC = 0.946, 95% CI: 0.902–0.989), indicating effective differentiation between low-grade and high-grade gliomas. (**B**) Precision–recall curve demonstrating strong performance in identifying high-grade glioma, with a high average precision (AP = 0.98) across recall levels. (**C**) Calibration curve: Predicted probabilities are compared with observed event frequencies; the calibration analysis yielded a slope of 1.29 and an intercept of 0.38, indicating a deviation from perfect calibration. (**D**) Confusion matrix: using the predefined optimal threshold P(HGG) = 0.38, the model achieved an accuracy of 88.3%, sensitivity of 92.2%, and specificity of 76.7%. (**E**) Decision curve analysis: the model showed a higher net benefit than the treat-all and treat-none strategies across threshold probabilities of approximately 0.05–0.75.

The final Random Forest model was rigorously evaluated on the internal validation set (*n* = 120) to assess its discriminative ability, calibration, and clinical utility. The results demonstrated that the model performed stably and exhibited strong discriminative power on unseen data.;

In terms of discriminative ability, the model achieved an area under the receiver operating characteristic curve (AUC) of 0.946 (95% CI: 0.902–0.989), indicating excellent discriminative ability in distinguishing HGG from LGG (Figure 4A). The precision-recall (PR) curve reached an average precision of 0.98, further illustrating the model’s high recognition accuracy despite the imbalanced class distribution in the dataset (Figure 4B). The calibration curve demonstrated reasonable agreement between predicted and observed probabilities (calibration slope = 1.29, intercept = 0.38), although slight overestimation of risk was observed (Figure 4C). Using the predefined optimal probability threshold (P(HGG) = 0.38), the confusion matrix showed an overall accuracy of 88.3%, sensitivity of 92.2%, and specificity of 76.7%. The balanced accuracy was 84.5%, indicating stable classification performance across both classes under imbalanced conditions (Figure 4D). Decision curve analysis demonstrated that across threshold probabilities of approximately 0.05–0.75, the Random Forest model yielded a higher net benefit than both the treat-all and treat-none strategies, indicating potential clinical utility within this range (Figure 4E).

Overall, the Random Forest model demonstrated strong performance in the internal validation set, supporting its potential utility for non-invasive preoperative glioma grading.

### 3.5. Model Interpretability Analysis Based on SHAP

**Figure 5:**
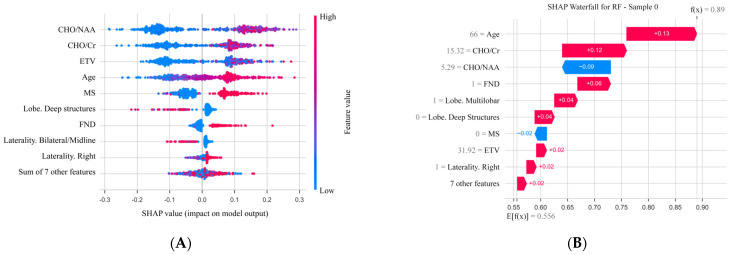
Interpretation of the random forest model using SHAP. (**A**) SHAP summary plot: features are ranked by mean absolute SHAP values. Each point represents a patient in the validation cohort, with SHAP values indicating the direction and magnitude of each feature’s contribution to the predicted probability of HGG (positive values increase the probability of HGG). (**B**) SHAP waterfall plot for an illustrative patient from the validation set: the plot decomposes the predicted probability (0.89) into feature-level contributions relative to the baseline model output (E[f(X)]). Key contributing features, including age (66 years) and Cho/Cr ratio (15.32), are shown as major drivers of the prediction in this example.

To elucidate the decision-making process of the Random Forest model, Shapley Additive exPlanations (SHAP) was used for post hoc interpretability analysis. The SHAP summary plot (Figure 5A) illustrates the relative importance and directional contribution of each feature.

Overall, the contributions of different features to the model output varied in magnitude, and the direction of the major contributing features aligned closely with existing clinical knowledge. Among the features, the Cho/NAA ratio was identified as the most influential feature, with higher values associated with positive SHAP values (red points), indicating increased probability of HGG. Similarly, elevated Cho/Cr ratio, increased patient age, larger ETV, and the presence of both MS and FND were associated with positive SHAP values. These findings were consistent with known clinical associations of high-grade glioma. Furthermore, other features, including the tumor laterality and lobar location also contributed to the final model output to varying degrees.

To further illustrate the decision-making process at the individual level, we present a SHAP waterfall plot for a randomly selected patient (Figure 5B). The baseline prediction for this patient (the overall mean predicted probability) was 0.556. After incrementally adding the positive and negative contributions of each feature, the model adjusted the patient’s predicted probability to 0.89 for HGG. In this case, advanced age (66 years) and a high Cho/Cr ratio (15.32) were the major positive contributors increasing the predicted risk, while the relatively low Cho/NAA ratio (5.29) acted as a negative contributor, offsetting influence. This visualization provides an interpretable decomposition of the individual prediction into feature-level contributions.

## 4. Discussion

Glioma represents the most prevalent primary malignant tumor of the central nervous system [1]. Despite advances in multimodal diagnosis and treatment, prognosis remains unsatisfactory due to aggressive biological behavior [2]. Accurate preoperative grading is therefore critical for surgical planning and adjuvant treatment selection under the Stupp protocol [26,27]. However, conventional grading relies on postoperative histopathology, which is invasive and may not adequately support preoperative decision-making [28]. In the present study, we developed and internally validated an interpretable machine-learning model integrating clinical variables, structural imaging features, and MRS-derived metabolic biomarkers for preoperative glioma grading. The results indicate that the model demonstrated strong discriminatory performance in the internal validation cohort. In addition, routinely available multimodal variables contributed meaningfully to prediction, and SHAP-based interpretation improved transparency of the decision process at both global and individual levels. These findings suggest that integrating accessible multimodal data with interpretable machine-learning may represent a feasible approach for preoperative glioma risk stratification in a single-center setting.

With an AUC of 0.946, our model achieved performance comparable to advanced radiomics-based approaches [29,30] and to studies reporting higher AUC values using complex image-processing pipelines [31]. This comparison is noteworthy because many previously published models depend on high-dimensional radiomic extraction, labor-intensive image preprocessing, or dedicated post-processing workflows, which may limit reproducibility and routine clinical implementation [30,31]. In addition, some prior investigations rely predominantly on single-modality features or focus on isolated clinical associations without integrated validation [16,32], highlighting the limited integration of multimodal information in existing approaches. In contrast, our model integrates clinically interpretable variables and routine imaging parameters within a unified framework, thereby offering a more pragmatic modeling strategy for exploratory preoperative assessment. The favorable performance of the Random Forest model may be attributed to its ability to capture complex nonlinear relationships and interactions among multimodal predictors. Given the heterogeneity of clinical symptoms, structural imaging features, and metabolic biomarkers, ensemble tree-based methods may offer advantages over simpler linear approaches in modeling such heterogeneous clinical-imaging data.

Among the selected predictors, MRS-derived metabolic ratios played a prominent role. Elevated Cho/NAA and Cho/Cr ratios were positively associated with high-grade classification, consistent with the biological interpretation that choline reflects membrane turnover, NAA represents neuronal integrity, and creatine serves as a relatively stable metabolic reference [33,34,35]. These metabolic alterations suggest increased tumor proliferation and neuronal disruption, aligning with prior findings [35]. However, variability in MRS acquisition parameters, voxel placement, and scanner differences across institutions may affect reproducibility. Broader validation under heterogeneous acquisition conditions is therefore warranted. Structural imaging features, including midline shift (MS) and enhancing tumor volume (ETV), also contributed meaningfully. HGG typically exhibits rapid growth, substantial mass effect, and blood–brain barrier disruption, leading to increased MS and ETV. Tumor volume in this study was estimated using the ellipsoid approximation formula, which reflects routine clinical practice but may introduce measurement bias compared with full volumetric segmentation. Future incorporation of precise volumetric techniques may further refine predictive performance. Clinical variables such as age and focal neurological deficits (FND) additionally strengthened model prediction. Older age is well recognized as being associated with higher-grade glioma and poorer prognosis [36], while FND likely reflects aggressive infiltrative growth and vasogenic edema [37,38,39]. Tumor laterality and lobar location also demonstrated spatial associations with grading risk [40,41,42], although the underlying biological mechanisms warrant further exploration.

Importantly, the model outputs an individualized probability of HGG (P(HGG)), allowing clinicians to interpret risk on a continuous scale rather than relying solely on binary classification. For the purpose of calculating classification-based performance metrics, a predefined cutoff (P(HGG) = 0.38) was applied, yielding a sensitivity of 92.2% and specificity of 76.7% in the validation cohort. From a clinical perspective, this probabilistic output may be more informative than a simple dichotomous label, as it can assist clinicians in integrating model estimates with anatomical location, symptom burden, and other perioperative considerations. This may provide additional support for preoperative risk assessment and clinical judgment. Nevertheless, the current model should be regarded as a decision-support tool for exploratory evaluation rather than a standalone basis for treatment selection. Decision curve analysis further demonstrated net benefit across threshold probabilities of approximately 0.05–0.75, supporting potential clinical utility within this range. Although discrimination was strong, calibration analysis indicated mild overestimation of predicted probabilities (slope = 1.29, intercept = 0.38), possibly related to the relatively high HGG prevalence in the cohort. External validation and recalibration may further improve probability accuracy.

Several aspects of this study may strengthen its practical relevance. First, the model integrates multimodal yet routinely obtainable preoperative data without requiring complex radiomics extraction or dedicated segmentation software. Second, the dual feature-selection strategy enhances robustness by retaining consistently supported predictors. Third, incorporation of SHAP-based interpretability allows transparent visualization of feature contributions, helping bridge the gap between algorithmic prediction and clinical reasoning. These features may improve methodological transparency and facilitate future external testing.

This study has several limitations. Its single-center retrospective design may introduce selection bias, and although the overall sample size met methodological requirements, the relatively smaller LGG subgroup may influence performance stability. The model has not yet undergone external validation, and generalizability across diverse populations, imaging platforms, and acquisition protocols remains to be established. Moreover, potential dataset shift arising from variations in imaging protocols, scanner types, and patient populations across institutions may affect model performance. Furthermore, the classification threshold was determined based on the F1-score during model evaluation rather than within a cross-validation or external validation framework, which may introduce a degree of optimistic bias. Additionally, reliance on MRS parameters and approximate volumetric estimation may limit reproducibility in settings where standardized protocols are unavailable. Prospective multicenter validation, recalibration, and evaluation of real-world clinical impact and cost-effectiveness are warranted to further define the model’s practical applicability.

This study has several limitations. Its single-center retrospective design may introduce selection bias, and although the overall sample size met methodological requirements, the relatively smaller LGG subgroup may influence performance stability. The model has not yet undergone external validation, and its generalizability across different populations, imaging platforms, and acquisition protocols remains to be established. Potential dataset shift related to variations in imaging protocols, scanner types, and patient characteristics across institutions may also affect model performance. The classification threshold was determined based on the F1-score during model evaluation rather than within a cross-validation or external validation framework, which may introduce a degree of optimistic bias. In addition, reliance on MRS parameters and approximate volumetric estimation may limit reproducibility in settings without standardized imaging protocols. Prospective multicenter validation, model recalibration, and evaluation of real-world clinical impact and cost-effectiveness are warranted to further define the model’s practical applicability.

## 5. Conclusions

This study developed and internally validated a multimodal machine-learning model for non-invasive preoperative glioma grading by integrating clinical features, structural imaging, and MRS-derived metabolic biomarkers. The model showed good discrimination and acceptable calibration in the internal validation cohort and provided individualized probability estimates for glioma grading, supporting non-invasive preoperative assessment and risk stratification. However, given the single-center retrospective design and the lack of external or prospective validation, clinical application remains premature, and further validation in independent cohorts is required.

## Figures and Tables

**Table 1:** (**A**) Baseline clinical characteristics of all participants. (**B**) Baseline imaging structure characteristics of all participants. (**C**) Baseline MRS parameters of all participants.

Variable	Total	LGG	HGG	*p*-Value	Chi-Squared
	N = 400	N = 100	N = 300		
**(A) Clinical characteristics**					
Gender, n (%)				0.906	0.014
Male	242 (60.5)	60 (60)	182 (60.7)		
Female	158 (39.5)	40 (40)	118 (39.3)		
Age, Median (IQR)	53.0 (43.8, 62.0)	42.5 (29.8, 51.2)	55.0 (47.0, 64.0)	<0.001	61.759
Hypertension, n (%)				0.102	2.675
No	326 (81.5)	87 (87)	239 (79.7)		
Yes	74 (18.5)	13 (13)	61 (20.3)		
Diabetes, n (%)				0.063	3.46
No	377 (94.2)	98 (98)	279 (93)		
Yes	23 (5.8)	2 (2)	21 (7)		
Smoking, n (%)				0.371	0.801
No	309 (77.2)	74 (74)	235 (78.3)		
Yes	91 (22.8)	26 (26)	65 (21.7)		
Drinking alcohol, n (%)				0.876	0.024
No	334 (83.5)	83 (83)	251 (83.7)		
Yes	66 (16.5)	17 (17)	49 (16.3)		
IIP, n (%)				0.686	0.163
No	197 (49.2)	51 (51)	146 (48.7)		
Yes	203 (50.7)	49 (49)	154 (51.3)		
Seizure, n (%)				<0.001	23.171
No	331 (82.8)	67 (67)	264 (88)		
Yes	69 (17.2)	33 (33)	36 (12)		
FND, n (%)				<0.001	20.802
No	270 (67.5)	86 (86)	184 (61.3)		
Yes	130 (32.5)	14 (14)	116 (38.7)		
CI, n (%)				0.67	0.181
No	368 (92.0)	93 (93)	275 (91.7)		
Yes	32 (8.0)	7 (7)	25 (8.3)		
CS, n (%)				0.263	Fisher
No	382 (95.5)	98 (98)	284 (94.7)		
Yes	18 (4.5)	2 (2)	16 (5.3)		
Duration, Median (IQR)	1.0 (0.3, 2.0)	1.0 (0.3, 4.0)	0.7 (0.3, 2.0)	0.166	1.921
BMI, Median (IQR)	23.7 (21.5, 26.1)	24.0 (21.3, 26.7)	23.5 (21.7, 26.0)	0.840	0.041
KPS ≥ 70, n (%)				0.001	10.73
No	120 (30.0)	17 (17)	103 (34.3)		
Yes	280 (70.0)	83 (83)	197 (65.7)		
**(B) Imaging characteristics**					
Laterality, n (%)				<0.001	14.782
Bilateral/Midline	22 (5.5)	13 (13)	9 (3)		
Left	176 (44.0)	43 (43)	133 (44.3)		
Right	202 (50.5)	44 (44)	158 (52.7)		
Lobe, n (%)				0.002	Fisher
Multilobar	72 (18.0)	13 (13)	59 (19.7)		
Frontal Lobe	162 (40.5)	43 (43)	119 (39.7)		
Temporal Lobe	84 (21.0)	15 (15)	69 (23)		
Parietal Lobe	40 (10.0)	8 (8)	32 (10.7)		
Occipital Lobe	7 (1.8)	1 (1)	6 (2)		
Deep Structures	30 (7.5)	17 (17)	13 (4.3)		
Posterior Fossa	5 (1.2)	3 (3)	2 (0.7)		
MS, n (%)				<0.001	55.505
No	195 (48.8)	81 (81)	114 (38)		
Yes	205 (51.2)	19 (19)	186 (62)		
Unifocal, n (%)				0.092	2.831
No	21 (5.2)	2 (2)	19 (6.3)		
Yes	379 (94.8)	98 (98)	281 (93.7)		
ETV, Median (IQR)	57.8 (20.0, 83.0)	17.7 (12.7, 27.4)	73.8 (33.7, 88.3)	<0.001	87.521
**(C) MRS parameters**					
CHO/NAA, Median (IQR)	12.2 (5.1, 21.5)	3.7 (2.2, 6.4)	16.6 (8.1, 24.7)	<0.001	99.311
CHO/Cr, Median (IQR)	4.3 (2.6, 8.6)	2.4 (1.8, 3.0)	5.8 (3.4, 8.8)	<0.001	95.702
NAA/Cr, Median (IQR)	0.4 (0.2, 0.5)	0.5 (0.4, 0.8)	0.3 (0.2, 0.5)	<0.001	24.277

**Table 2:** (**A**) Univariate comparison of clinical candidate predictors between LGG and HGG in the training cohort. (**B**) Univariate comparison of structural imaging candidate predictors between LGG and HGG in the training cohort. (**C**) Univariate comparison of MRS candidate predictors between LGG and HGG in the training cohort.

Variables	Total (*n* = 280)	LGG (*n* = 69)	HGG (*n* = 211)	*p*-Value	Chi-Squared
**(A) Clinical characteristics**					
Gender, n (%)				0.416	0.662
Male	171 (61.1)	45 (65.2)	126 (59.7)		
Female	109 (38.9)	24 (34.8)	85 (40.3)		
Age, Median (IQR)	52.0 (43.8, 63.0)	43.0 (32.0, 51.0)	56.0 (47.0, 64.0)	<0.001	43.226
Hypertension, n (%)				0.137	2.212
No	231 (82.5)	61 (88.4)	170 (80.6)		
Yes	49 (17.5)	8 (11.6)	41 (19.4)		
Diabetes, n (%)				0.009	Fisher
No	263 (93.9)	69 (100)	194 (91.9)		
Yes	17 (6.1)	0 (0)	17 (8.1)		
Smoking, n (%)				0.214	1.545
No	218 (77.9)	50 (72.5)	168 (79.6)		
Yes	62 (22.1)	19 (27.5)	43 (20.4)		
Drinking alcohol, n (%)				0.19	1.714
No	237 (84.6)	55 (79.7)	182 (86.3)		
Yes	43 (15.4)	14 (20.3)	29 (13.7)		
IIP, n (%)				0.826	0.048
No	129 (46.1)	31 (44.9)	98 (46.4)		
Yes	151 (53.9)	38 (55.1)	113 (53.6)		
Seizure, n (%)				<0.001	17.882
No	230 (82.1)	45 (65.2)	185 (87.7)		
Yes	50 (17.9)	24 (34.8)	26 (12.3)		
FND, n (%)				<0.001	18.242
No	189 (67.5)	61 (88.4)	128 (60.7)		
Yes	91 (32.5)	8 (11.6)	83 (39.3)		
CI, n (%)				0.464	0.537
No	258 (92.1)	65 (94.2)	193 (91.5)		
Yes	22 (7.9)	4 (5.8)	18 (8.5)		
CS, n (%)				0.46	Fisher
No	270 (96.4)	68 (98.6)	202 (95.7)		
Yes	10 (3.6)	1 (1.4)	9 (4.3)		
Duration, Median (IQR)	1.0 (0.3, 2.0)	1.0 (0.2, 4.0)	1.0 (0.3, 2.0)	0.32	0.989
BMI, Median (IQR)	23.4 (21.6, 26.1)	24.2 (21.5, 26.9)	23.3 (21.6, 25.5)	0.251	1.319
KPS ≥ 70, n (%)				0.017	5.744
No	85 (30.4)	13 (18.8)	72 (34.1)		
Yes	195 (69.6)	56 (81.2)	139 (65.9)		
**(B) Imaging characteristics**	**Total (*n* = 280)**	**LGG (*n* = 69)**	**HGG (*n* = 211)**	***p*-Value**	**Chi-Squared**
Laterality, n (%)				0.006	Fisher
Bilateral/Midline	17 (6.1)	10 (14.5)	7 (3.3)		
Left	115 (41.1)	25 (36.2)	90 (42.7)		
Right	148 (52.9)	34 (49.3)	114 (54)		
Lobe, n (%)				<0.001	Fisher
Multilobar	52 (18.6)	9 (13)	43 (20.4)		
Frontal Lobe	111 (39.6)	32 (46.4)	79 (37.4)		
Temporal Lobe	64 (22.9)	11 (15.9)	53 (25.1)		
Parietal Lobe	26 (9.3)	3 (4.3)	23 (10.9)		
Occipital Lobe	4 (1.4)	0 (0)	4 (1.9)		
Deep Structures	21 (7.5)	13 (18.8)	8 (3.8)		
Posterior Fossa	2 (0.7)	1 (1.4)	1 (0.5)		
MS, n (%)				<0.001	30.756
No	138 (49.3)	54 (78.3)	84 (39.8)		
Yes	142 (50.7)	15 (21.7)	127 (60.2)		
Unifocal, n (%)				0.742	Fisher
No	13 (4.6)	2 (2.9)	11 (5.2)		
Yes	267 (95.4)	67 (97.1)	200 (94.8)		
ETV, Median (IQR)	51.6 (18.1, 83.0)	17.7 (12.7, 22.7)	75.0 (32.2, 91.7)	<0.001	68.698
**(C) MRS parameters**	**Total (*n* = 280)**	**LGG (*n* = 69)**	**HGG (*n* = 211)**	***p*-Value**	**Chi-Squared**
CHO/NAA, Median (IQR)	11.6 (5.0, 22.1)	3.6 (2.1, 6.4)	16.1 (7.9, 24.7)	<0.001	60.334
CHO/Cr, Median (IQR)	4.3 (2.6, 8.7)	2.3 (1.8, 3.0)	5.8 (3.1, 8.8)	<0.001	58.042
NAA/Cr, Median (IQR)	0.4 (0.2, 0.6)	0.5 (0.3, 0.8)	0.4 (0.2, 0.5)	<0.001	14.285

**Table 3:** Multivariate regression analysis of the training set.

Variable	OR (95%CI)	*p*-Value
**Age**	1.08 (1.06~1.11)	<0.001
**Seizure**		
No	1 (Ref)	
Yes	0.26 (0.14~0.5)	<0.001
**FND**		
No	1 (Ref)	
Yes	4.94 (2.25~10.86)	<0.001
**Laterality**		
Bilateral/Midline	1 (Ref)	
Left	5.14 (1.78~14.88)	0.003
Right	4.79 (1.69~13.54)	0.003
**Lobe**		
Multilobar	1 (Ref)	
Frontal Lobe	0.52 (0.23~1.18)	0.118
Temporal Lobe	1.01 (0.38~2.66)	0.986
Parietal Lobe	1.6 (0.4~6.52)	0.508
Occipital Lobe	Not estimable due to sparse data	
Deep Structures	0.13 (0.04~0.4)	<0.001
Posterior Fossa	0.21 (0.01~3.67)	0.284
**KPS ≥ 70**		
No	1 (Ref)	
Yes	0.45 (0.23~0.87)	0.018
**MS**		
No	1 (Ref)	
Yes	5.44 (2.88~10.27)	<0.001
**ETV**	1.03 (1.02~1.04)	<0.001
**CHO/NAA**	1.04 (1.01~1.07)	0.004
**CHO/Cr**	1.2 (1.09~1.33)	<0.001
**NAA/Cr**	0.87 (0.67~1.13)	0.3

## Data Availability

The original contributions presented in this study are included in the article. Further inquiries can be directed to the corresponding authors.

## Supplementary Materials

The following supporting information can be downloaded at: https://www.mdpi.com/article/10.3390/cancers18081204/s1, Table S1: Performance metrics for the evaluated models, including Accuracy, AUC, Recall, Precision, F1-score, Cohen’s Kappa, Matthews Correlation Coefficient (MCC), Log Loss, Brier Score, and Training Time (seconds).

## Abbreviations

The following abbreviations are used in this manuscript:

AUC	Area Under the Curve
Brier	Brier Score
CI	Cognitive and psychiatric symptoms
CS	Cerebellar symptoms
DCA	Decision-curve analysis
ETV	Enhanced tumor volume
FND	Focal neurological deficits
F1	F1-Score
HGG	High-grade glioma
IIP	Intracranial increased pressure
Kappa	Cohen’s Kappa
LGG	Low-grade glioma
MS	Midline shift
MCC	Matthews Correlation Coefficient
Prec.	Precision
ROC	Receiver operating characteristic
TT(Sec)	Training Time (Seconds)
