# Supplementary material for: An Interpretable Multimodal Machine-Learning Model for Non-Invasive Preoperative Glioma Grading
Source: Cancers (Basel). 2026 Apr 10;18(8):1204. doi: 10.3390/cancers18081204 (PMC13114312; doi:10.3390/cancers18081204)
Supplement: Supplementary file 1 [file cancers-18-01204-s001.zip › cancers-4210024-supplementary.pdf]

Supplementary Table 1: Performance metrics for the evaluated models, including Accuracy, AUC, Recall, Precision, F1-score, Cohen's Kappa, Matthews Correlation Coefficient (MCC), Log Loss, Brier Score, and Training Time (seconds).

Model	Accuracy	AUC	Recall	Prec.	F1	Kappa	MCC	Log Loss	Brier	TT (Sec)
Random Forest Classifier	0.8766	0.9189	0.9333	0.9076	0.9201	0.6665	0.6688	4.3767	0.1214	0.204
Ada Boost Classifier	0.8679	0.9126	0.9	0.9218	0.9101	0.66	0.6639	4.7629	0.1321	0.166
CatBoost Classifier	0.8714	0.9105	0.9143	0.9141	0.9142	0.658	0.6582	4.6342	0.1286	2.476
Gradient Boosting Classifier	0.8679	0.9071	0.9095	0.9135	0.9112	0.6524	0.6542	4.7629	0.1321	0.198
Extra Trees Classifier	0.8643	0.9053	0.9238	0.8987	0.9109	0.6261	0.6281	4.8916	0.1357	0.18
Extreme Gradient Boosting	0.8607	0.9041	0.9143	0.9023	0.9079	0.6217	0.6245	5.0004	0.1393	0.15
Light Gradient Boosting Machine	0.8607	0.9017	0.919	0.8985	0.9084	0.6177	0.6201	5.0004	0.1393	0.128
MLP Classifier	0.8357	0.8905	0.8667	0.9104	0.8876	0.5823	0.5862	5.9215	0.1643	0.498
K Neighbor Classifier	0.8143	0.8861	0.8238	0.9209	0.8685	0.5542	0.5668	6.6938	0.1857	0.128
Logistic Regression	0.8036	0.8735	0.819	0.9146	0.8615	0.522	0.5372	7.08	0.1964	0.14
Linear Discriminant Analysis	0.8	0.8724	0.8143	0.9102	0.8587	0.5189	0.5302	7.2087	0.2	0.112
Ridge Classifier	0.8	0.8721	0.8143	0.91	0.8588	0.5185	0.5294	7.2087	0.2	0.12
SVM - Linear Kernel	0.7964	0.8235	0.8524	0.8845	0.8598	0.4557	0.4961	7.3375	0.2036	0.122
Gaussian Process Classifier	0.8321	0.8168	0.8381	0.9313	0.8814	0.5963	0.6081	6.0502	0.1679	0.254
Naive Bayes	0.6357	0.8048	0.581	0.8922	0.6813	0.312	0.3537	13.1302	0.3643	0.12
Decision Tree Classifier	0.8071	0.7667	0.8476	0.8893	0.8666	0.5174	0.5239	6.9513	0.1929	0.122
Dummy Classifier	0.25	0.5	0.0	0.0	0.0	0.0	0.0	27.0327	0.75	0.116
